# Clinical and imaging features of primary thyroid MALT lymphoma

**DOI:** 10.3389/fonc.2025.1498609

**Published:** 2025-05-08

**Authors:** Liming Xiao, Ziyi Zhao, Li Zhou, Jiao Yan, Danling Luo, Fucen Liu, Qiaolin Zhou, Dan Huang

**Affiliations:** ^1^ Department of Nuclear Medicine, Mianyang Central Hospital, School of Medicine, University of Electronic Science and Technology of China, Mianyang, China; ^2^ National Health Commission (NHC) Key Laboratory of Nuclear Technology Medical Transformation (Mianyang Central Hospital), Mianyang, China; ^3^ Department of Pathology, Mianyang Central Hospital, School of Medicine, University of Electronic Science and Technology of China, Mianyang, China; ^4^ Department of Ultrasound, Mianyang Central Hospital, School of Medicine, University of Electronic Science and Technology of China, Mianyang, China; ^5^ Department of Hematology, Mianyang Central Hospital, School of Medicine, University of Electronic Science and Technology of China, Mianyang, China

**Keywords:** primary thyroid lymphoma, mucosa-associated lymphoid tissue lymphoma, case report, system review, literature review

## Abstract

Primary thyroid lymphoma is a rare hematologic malignancy of the thyroid gland, accounting for approximately 5% of all malignant thyroid tumors. The most common pathological type is B-cell-derived non-Hodgkin’s lymphoma, mainly diffuse large B-cell lymphoma, followed by mucosa-associated lymphoid tissue lymphoma and mixed types. The clinical and radiographic characteristics of primary thyroid lymphoma are non-specific, often leading to misdiagnosis as thyroiditis and a delay in treatment. A 60-year-old woman was referred to our hospital with neck swelling that had persisted for a week. Histopathological findings of a thyroid biopsy revealed mucosa-associated lymphoid tissue lymphoma. Bone marrow examination revealed atypical lymphocytes on myelograms. [^18^F]FDG PET/CT images showed increased [^18^F]FDG uptake in both lobes of the thyroid gland and the cervical lymph nodes. The patient was diagnosed with stage IV primary thyroid mucosa-associated lymphoid tissue lymphoma. The patient subsequently received four cycles of R-CEOP chemotherapy and remained under follow-up. Due to the rarity of this case, we conducted a systematic literature review to better understand the disease and improve timely diagnosis and treatment.

## Introduction

Primary thyroid lymphoma (PTL) is a rare hematologic malignancy of the thyroid gland, accounting for approximately 5% of all malignant thyroid tumors (1). The most common pathological type is B-cell-derived non-Hodgkin’s lymphoma, mainly diffuse large B-cell lymphoma (DLBCL) (70%), followed by mucosa-associated lymphoid tissue (MALT) lymphoma (15–30%) and mixed types (2, 3). PTL is more prevalent in older women (4). Moreover, its clinical and radiographic characteristics are non-specific. The most common clinical manifestation is a rapidly enlarging (<6 months) neck mass, often combined with cervical lymph node enlargement or compression symptoms, such as dyspnea and dysphagia (2, 5), especially among patients with a background of Hashimoto’s thyroiditis (HT) (6). We report a case of primary MALT lymphoma of the thyroid gland. Due to the rarity of this case, we conducted a systematic review of the literature on PTL to enhance clinicians’ understanding of the disease and improve diagnosis and treatment strategies.

## Case presentation

**Figure 1:**
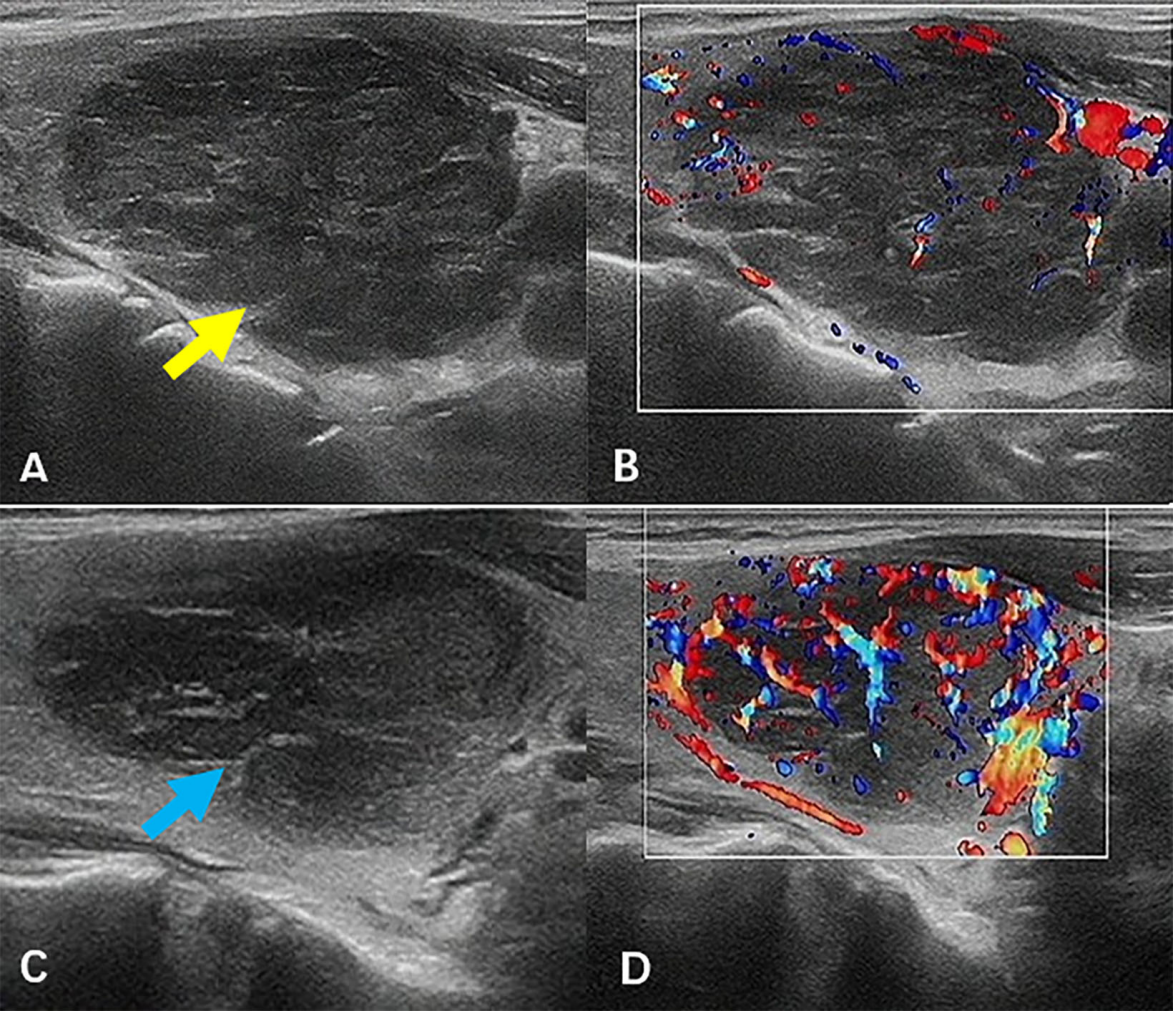
Ultrasound images. Hypoechoic lesions were evident in both thyroid gland lobes (**A**, **B**, the left lobe, yellow arrow; **C**, **D**, the right lobe, blue arrow), with a clear boundary, a regular shape, and an uneven internal echo. CDFI revealed abundant blood flow signals in and around the lesions. CDFI, color doppler flow imaging.

A 60-year-old woman presented with neck swelling that had persisted for 1 week. The patient was previously in good health and had no family history of thyroid disease. Physical examination revealed neck swelling and bilateral thyroid enlargement. Enlarged superficial lymph nodes were palpable in the neck, exhibiting a hard texture and difficult to move. Laboratory examinations revealed that the anti-thyroglobulin antibody levels were above the normal range. The patient had mild anemia and normal tumor markers, renal function, and coagulation function. Key laboratory results are presented in **Table 1**. **Figure 1** illustrates the ultrasound images. Ultrasound images revealed a thyroid gland with abnormal morphology, an unsmooth capsule, and uneven thickening of parenchymal echoes. Color doppler flow imaging (CDFI) showed increased blood flow signals. Hypoechoic lesions were evident in both thyroid gland lobes, with a clear boundary, a regular shape, and an uneven internal echo. The hypoechoic lesion in the left lobe was about 4.3×3.1×2.8 cm in size, and that in the right lobe was about 2.8×2.6×1.8 cm in size. CDFI revealed abundant blood flow signals in and around the lesions (**Figures 1A, B**, the left lobe, yellow arrow; **Figures 1C, D**, the right lobe, blue arrow). According to the ACR Thyroid Imaging, Reporting and Data System (TI-RADS) (7), the hypoechoic lesions were awarded four points; hence, the risk levels were characterized as TR4. Multiple lymph nodes were apparent in the bilateral neck (level IV), and the boundary between the cortex and medulla of the lymph nodes was unclear.

**Table 1:** Key laboratory results.

Laboratory examination	Result		Reference Range	Unit
Thyroid function				
Free Triiodothyronine (FT3)	2.41		1.80–4.20	pg/mL
Free Thyroxine (FT4)	1.1		0.87–1.85	ng/dL
Thyroid Stimulating Hormone (TSH)	12.964	↑	0.350–5.100	μIU/mL
Anti-Thyroglobulin Antibody (ATG)	13.03	↑	≤4	IU/mL
Anti-Thyroid Peroxidase Antibody (A-TPO)	0.31		≤9.0	IU/mL
Thyroid-stimulating hormone receptor antibody (TRAb)	<0.800		<1.220	U/L
Complete blood count				
White Blood Cell Count (WBC)	4.09		3.50–9.50	×10^9^/L
Red Blood Cell Count (RBC)	3.61	↓	3.80–5.10	×10^12^/L
Hemoglobin (HGB)	107	↓	130–175	g/L
Platelet Count (PLT)	96	↓	100–300	×10^9^/L
Liver function				
Alanine Aminotransferase (ALT)	14		7–40	U/L
Aspartate Aminotransferase (AST)	17		13–35	U/L
Gamma-Glutamyl Transferase (GGT)	11		7–45	U/L
Alkaline Phosphatase (ALP)	95		50–135	U/L
Lactate Dehydrogenase (LDH)	268	↑	120–250	U/L
Total protein (TP)	68.49		65.00–85.00	g/L
Albumin (ALB)	39.37	↓	40.00–55.00	g/L
Globulin (GLB)	29.12		20.00–40.00	g/L

↑, above reference range; ↓, below reference range.

**Figure 2:**
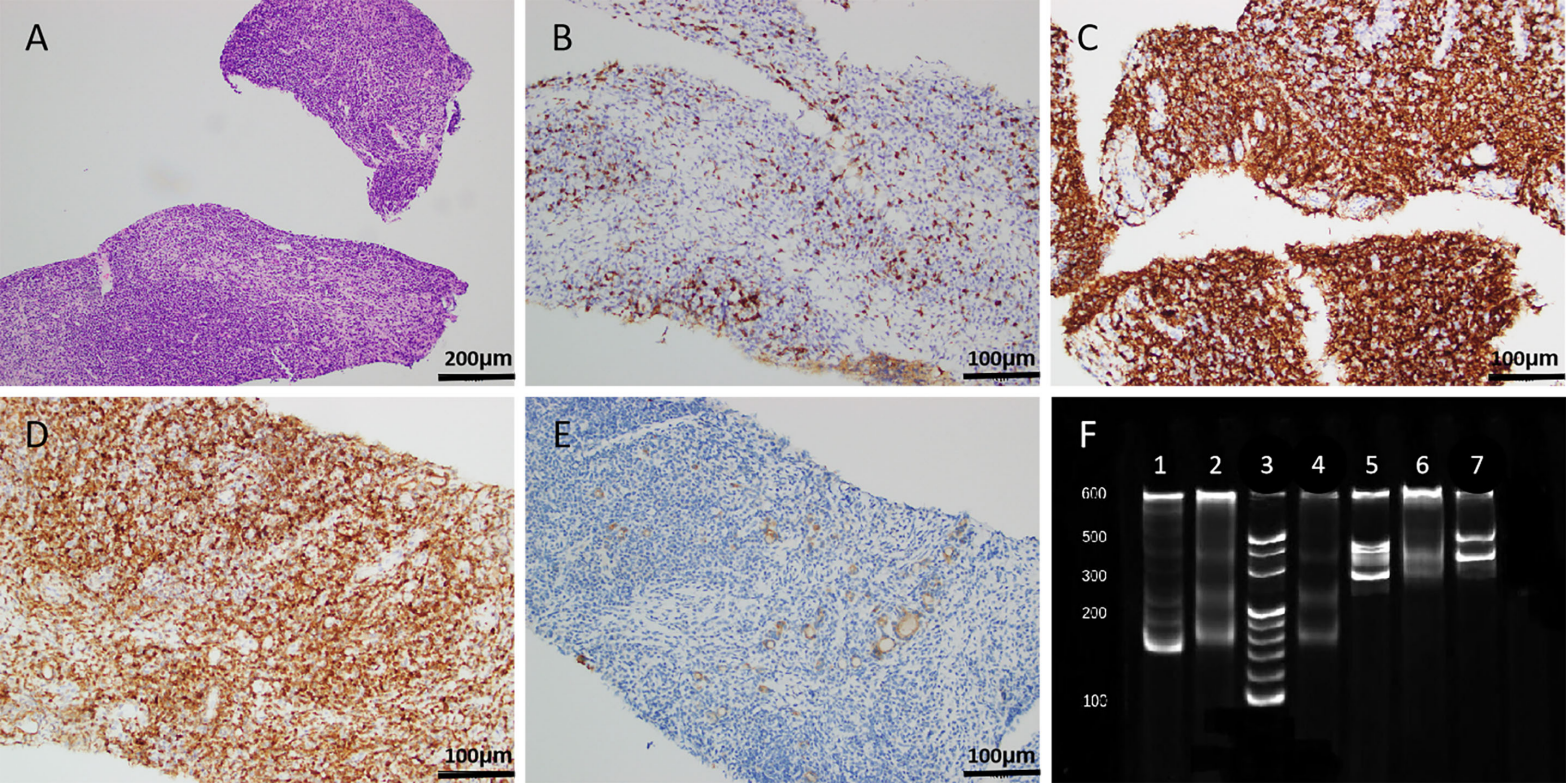
Histopathological results. (**A**, HE staining, magnification ×100) The thyroid tissue was infiltrated by numerous lymphocytes and plasma cells. (**B–E**, magnification ×200) Immunohistochemical analysis showed negative staining for CD5 **(B)** and positive staining for CD20 **(C)**, BcL-2 (approximately 80%, **D**), and P-CK **(E)**. **(F)** Immunoglobulin gene rearrangement demonstrated IGKB positivity (lane 1, positive control for IGKA; lane 2, negative control for IGKA; lane 3, marker; lane 4, IGKA; lane 5, positive control for IGKB; lane 6, negative control for IGKB; lane 7, IGKB). HE, haematoxylin and eosin; P-CK, Pan-Cytokeratin; IGKA, immunoglobulin kappa light chain allele A; IGKB, immunoglobulin kappa light chain allele B.

Subsequently, the patient underwent an ultrasound-guided thyroid core needle biopsy (CNB). Pathological findings revealed that the thyroid tissue was infiltrated by numerous lymphocytes and plasma cells [**Figure 2A**, haematoxylin and eosin (HE) staining, magnification ×100]. Immunohistochemical analysis showed negative staining for CD5 (**Figure 2B**, magnification ×200) and positive staining for CD20, BcL-2 (approximately 80%), and Pan-Cytokeratin (P-CK) (**Figures 2C–E**, magnification ×200). Moreover, immunoglobulin gene rearrangement demonstrated immunoglobulin kappa light chain allele B (IGKB) positivity (**Figure 2F**). Eventually, the patient was diagnosed with MALT lymphoma through a combination of histology, immunohistochemistry, and immunoglobulin gene rearrangement. Bone marrow morphology revealed atypical lymphocytes on myelograms.

**Figure 3:**
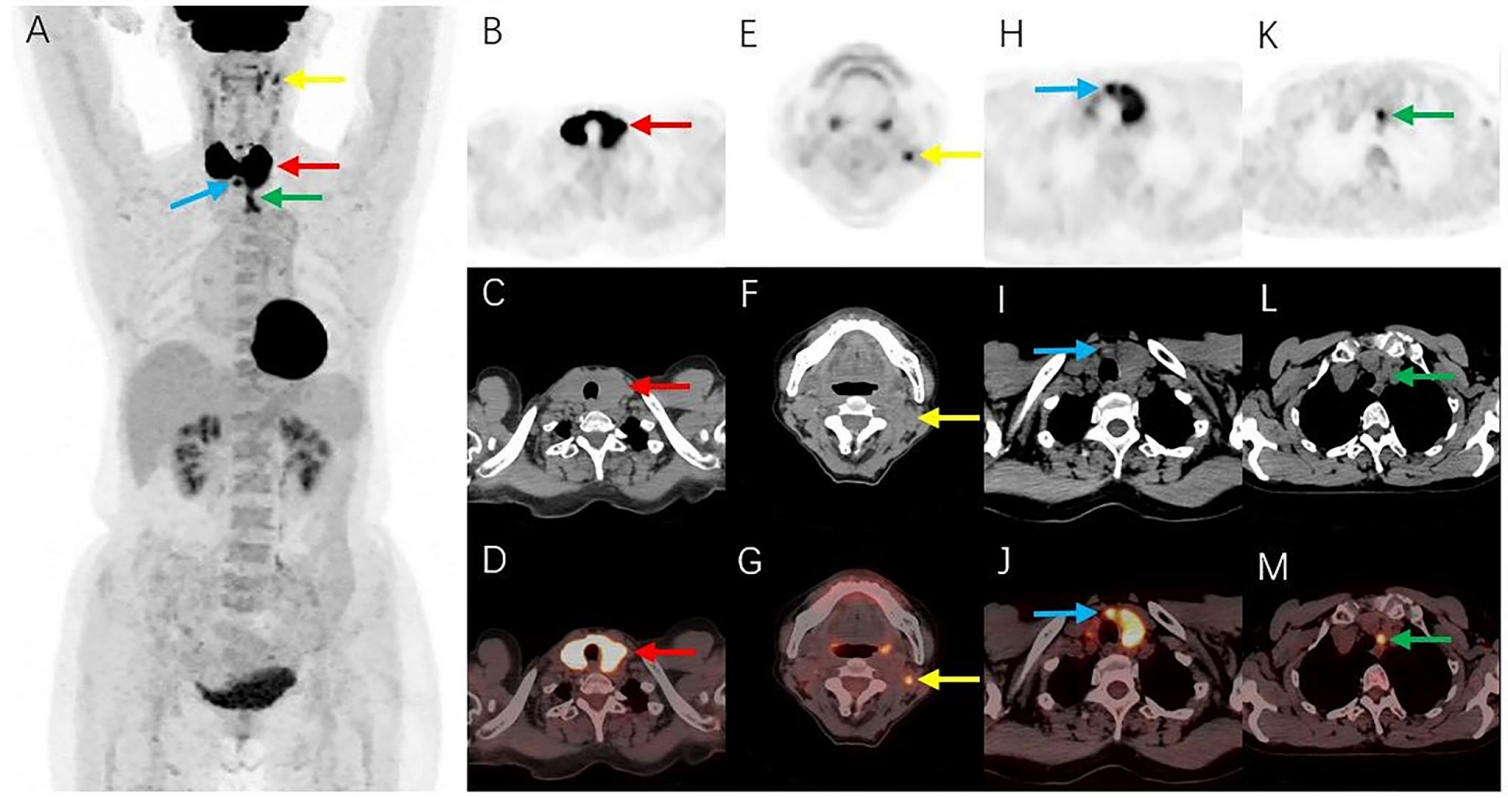
[^18^F]FDG PET/CT images. **(A)** Maximum intensity projections showed intense FDG uptake in both thyroid gland lobes (red arrow). Localized FDG uptake was also observed in the left level IIa (yellow arrow), level VI (blue arrow), and level VII (green arrow) of the neck. **(B–D)** Axial images (**B**, PET images; **C**, CT images; **D**, fusion images) showed intense FDG uptake in both thyroid gland lobes (red arrows), with diffuse enlargement and reduced density of both lobes on a corresponding CT scan. **(E–M)** Transverse PET **(E, H, K)**, CT **(F, I, L)**, and fusion images **(G, J, M)** showed lymph nodes with moderate FDG uptake in the left level IIa (yellow arrows), level VI (blue arrows), and level VII (green arrows) of the neck. [^18^F]FDG, 2-Deoxy-2-[^18^F]fluoro-D-glucose; PET/CT, positron emission tomography/computed tomography.

2-Deoxy-2-[^18^F]fluoro-D-glucose ([^18^F]FDG) positron emission tomography/computed tomography (PET/CT) was performed for pre-treatment staging. Maximum density projections of the anteroposterior positions (**Figure 3A**) showed intense [^18^F]FDG uptake in both thyroid gland lobes. Localized [^18^F]FDG uptake could also be observed in the left level IIa, level VI, and level VII of the neck. Axial images (**Figure 3B**, PET images; **Figure 3C**, CT images; **Figure 3D**, fusion images) revealed intense [^18^F]FDG uptake with a standardized uptake value maximum (SUVmax) of 19.6 in both thyroid gland lobes, with diffuse enlargement and reduced density of both lobes on a corresponding CT scan. Transverse PET (**Figures 3E, H, K**), CT (**Figures 3F, I, L**), and fusion images (**Figures 3G, J, M**) showed lymph nodes in the left level IIa, level VI, and level VII of the neck, with short diameters of approximately 0.5 cm, 0.8 cm, and 1.0 cm, respectively; moderate [^18^F]FDG uptake with an SUVmax of 5.3, 7.0, and 6.1, respectively; and suspected lymphoma involvement. No abnormal increase in [^18^F]FDG uptake was detected at other sites.

**Figure 4:**
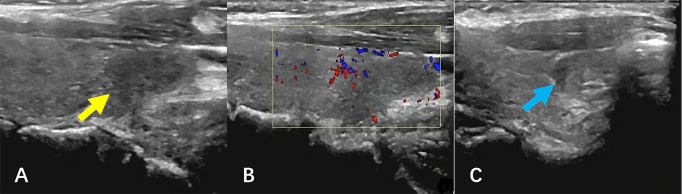
Ultrasound images after therapy. **(A, B)** The hypoechoic lesion was observed in the lower pole of the left lobe of the thyroid gland (yellow arrow), with an unclear boundary, a regular shape, and an uneven internal echo. CDFI revealed blood flow signals around the lesion. **(C)** A similar echo was seen in the middle pole of the right lobe of the thyroid gland (blue arrow), and CDFI showed no blood flow signal. CDFI, Color doppler flow imaging.

The patient subsequently received four cycles of R-CEOP immunochemotherapy: Rituximab 600 mg day 0, cyclophosphamide 1.2 g day 1, vincristine 2 mg day 1, epirubicin 110 mg day 1, and prednisone acetate 60 mg qd day 1–5. After therapy, the neck swelling disappeared, and the patient was in good condition. **Figure 4** shows the post-treatment ultrasound images. Ultrasonography revealed that the size and shape of the thyroid gland were restored to normal, the capsule was smooth, and the parenchymal echoes were slightly increased. CDFI revealed no abnormal blood flow signal. A hypoechoic lesion was observed in the lower pole of the left lobe of the thyroid gland, with an unclear boundary, a regular shape, and an uneven internal echo (**Figure 4A**, yellow arrow). The size range was about 1.5×1.1×0.6 cm. CDFI revealed blood flow signals around the lesion (**Figure 4B**). A similar echo pattern was seen in the middle pole of the right lobe of the thyroid gland, with a range of about 0.8×0.6×0.4 cm (**Figure 4C**, blue arrow). The patient remains under close follow-up and continues to be in remission.

## Systematic literature review

We searched the latest reports on this condition published in the PubMed database using the keyword combinations “thyroid lymphoma AND MALT” and “thyroid lymphoma AND mucosa-associated lymphoid tissue” from 2018 till June 2024. Initially, the reports were filtered and selected based on their titles and abstracts, followed by a comprehensive evaluation of their full text. We included only reports in the English language, focusing on imaging results, treatments, and outcomes. We excluded reviews, original articles, and reports without imaging results and complete medical history. Finally, we included 14 papers (8–21) in our analysis, which described 16 patients ranging in age from 43 to 86 years, with the majority being female (11/16).

The main symptom was neck swelling, and some patients also had cervical lymph node enlargement or compression symptoms, such as dyspnea and dysphagia. Ultrasound and CT findings were mainly characterized by thyroid enlargement and solid space-occupying lesions. Ultrasound showed hypoechoic lesions, and enhanced CT showed uneven enhancement. MRI of one patient showed a mass with low signal intensity on T1-weighted images and high signal intensity on T2-weighted images. All six patients had positive [^18^F]FDG uptake on [^18^F]FDG PET, and the SUVmax ranged from 6.0 to 26.82. Among the 16 patients with thyroid MALT lymphoma, 12 had thyroiditis, four had papillary thyroid carcinoma (PTC), one had thyroid tuberculosis, and five had other lymphomas or primary malignant tumors. The therapies included surgery, radiotherapy, chemotherapy, and immunochemotherapy, with surgery being the main treatment (12/16). Twelve patients responded well to treatment and showed remission changes. **Table 2** summarizes the details including imaging findings, treatments, and outcomes.

**Table 2:** Results of the systematic literature review on thyroid MALT lymphoma.

Reference (First author)	Year	Age/Gender	Ultrasound	CT (MRI)	PET	Treatment	Other related condition	Outcome
Tang R (8)	2024	81/F	N/A	Multiple enlarged lymph nodes	Multiple lymph nodes FDG-avid but Pentixafor-negative	Surgery	MALT lymphoma with highly aggressive B-cell lymphoma transformation	PD
Hsieh C-T (9)	2024	69/M	A diffuse, irregular hypoechoic lesion and reduced vascularity	A left thyroid tumor mass with central focal necrosis	FDG-avid masses over the left thyroid and lymph nodes	Immunochemotherapy (R-CHOP), radiotherapy	MALT lymphoma of adrenal glands with primary adrenal insufficiency	Remission
Zhang J (10)	2024	64/F	Thyroid enlargement	Significant enlargement of the thyroid gland	N/A	Surgery	HT, follicular thyroid adenoma, PTC	Remission
Munasinghe BM (11)	2024	50/M	A large multinodular goiter	N/A	N/A	Surgery, radiotherapy	Chronic thyroiditis, secondary brain metastases	PD (died of brain tumor complications)
Al Hassan MS (12)	2023	49/M	Large thyroid lobes, a small hypoechoic nodule	N/A	N/A	Surgery, radiotherapy	Painless subacute thyroiditis	Remission
Charoenngam N (13)	2023	67/F	Hypoechoic right-sided thyroid nodule lobe	N/A	Diffuse FDG uptake	Surgery	HT, hypothyroidism, breast cancer	Remission
Akbulut S (14)	2022	86/F	Bilateral multiple solid hypoechoic nodules	N/A	N/A	Surgery	Primary thyroid tuberculosis	Death of unknown cause
Kushwaha P (15)	2021	65/F	N/A	A homogeneous mass in the left lobe of the thyroid	N/A	Immunochemotherapy	HT, DLBCL of the bilateral submandibular glands, Multiple bone metastases	Died of pulmonary complications
Kesim S (16)	2021	66/F	N/A	N/A	Intense FDG uptake	Chemotherapy, radiotherapy	HT	Remission
Liu X-m (17)	2020	66/F	Inhomogeneous interior echoes and abundant blood flow signals	N/A	N/A	Radiotherapy	HT, hypothyroidism	Remission
Uchida N (18)	2020	67/F	N/A	CT: A poorly enhanced mass. MRI: low signal on T1-weighted images, high signal on T2-weighted images	N/A	Surgery	MALT lymphoma in ectopic mediastinal thyroid tissue, chronic thyroiditis	Remission
Acar N (19)	2019	51/F	Heterogeneous and hyperplastic thyroid gland	N/A	N/A	Surgery, chemotherapy, radiotherapy	HT	Remission
Lan X-B (20)	2018	57/M	Multinodular goiter	Mildly enhanced nodular foci	N/A	Surgery, radiotherapy	HT, PTC	Remission
		43/F	a calcific nodule, extensive thyromegaly	A large, inhomogeneous enhancement	N/A	Surgery	HT, PTC	Remission
		61/F	A hypoechoic mass and a small nodule	A large, inhomogeneous enhancement of the right thyroid gland	Slightly increased FDG uptake	Surgery, radiotherapy	HT, PTC, cervical carcinoma	Remission
Shrestha P (21)	2018	60/M	A large hypoechoic Mass	N/A	A hypermetabolic left thyroid mass and hypermetabolic thyroid lymph nodes	Surgery, radiotherapy	Basal cell carcinoma of the scalp	Remission

CT, computed tomography; MRI, magnetic resonance imaging; F, female; N/A, not available; [^18^F]FDG, 2-Deoxy-2-[^18^F]fluoro-D-glucose; HT, Hashimoto thyroiditis; MALT, Mucosa-associated lymphoid tissue; PD, Progressive disease; M, male; R-CHOP, rituximab, cyclophosphamide, adriamycin, vincristine and prednisone; PTC, papillary thyroid carcinoma; DLBCL, diffuse large B-cell lymphoma.

## Discussion

PTL is rare, accounting for approximately 5% of all malignant thyroid tumors (1). Most PTLs are B-cell-derived non-Hodgkin’s lymphoma, and the treatment and prognosis depend on the pathological type and stage of the tumor (2). The pathological types are mainly DLBCL, followed by MALT lymphoma and mixed types (3). **Table 3** provides an overview of thyroid DLBCL and MALT lymphoma. **Table 4** provides the differences between thyroid MALT lymphoma and other MALT lymphomas.

**Table 3:** Overview of thyroid DLBCL and MALT lymphoma.

Item	DLBCL	MALT lymphoma
Prevalence	>50%	10 ~ 23%
Morphological feature	More nodular type	More diffuse type
Clinical behaviour	More aggressive	Less aggressive
Histopathology	Large cells, monotonous lymphoid cells, lymphoepithelial lesions, decreased or absent colloid	Intermediate-sized cells, plasma cells, lymphoepithelial lesions, reactive lymphoid follicles
Treatment	Chemotherapy, radiation, targeted therapy	Radiation, chemotherapy, targeted therapy
Five-year survival rate	71 ~ 75%	96 ~ 100%

DLBCL, diffuse large B-cell lymphoma; MALT, mucosa-associated lymphoid tissue.

**Table 4:** Differences between thyroid MALT lymphoma and other MALT lymphomas.

Item	Thyroid MALT lymphoma	Gastric MALT lymphoma	Pulmonary MALT lymphoma	Salivary Gland MALT lymphoma	Cutaneous MALT lymphoma
Clinical Feature	Thyroid enlargement, possibly with compressive symptoms (e.g., dyspnea, dysphagia)	Epigastric pain, dyspepsia, nausea, vomiting, weight loss	Cough, dyspnea, chest pain, occasional hemoptysis	Salivary gland swelling, possibly with xerostomia or pain	Skin nodules, plaques, or ulcers
Surface Marker	CD20+, CD79a+, CD5-, CD10-	CD20+, CD79a+, CD5-, CD10-	CD20+, CD79a+, CD5-, CD10-	CD20+, CD79a+, CD5-, CD10-	CD20+, CD79a+, CD5-, CD10-
Treatment	Radiotherapy, chemotherapy, targeted therapy	Antibiotics (e.g., Helicobacter pylori eradication), radiotherapy, chemotherapy, targeted therapy	Radiotherapy, chemotherapy, targeted therapy	Radiotherapy, chemotherapy, targeted therapy	Local radiotherapy, surgical excision, targeted therapy
Prognosis	Favorable prognosis	Favorable prognosis	Favorable prognosis	Favorable prognosis	Excellent prognosis

MALT, mucosa-associated lymphoid tissue.

PTL is more prevalent in older females (4). Its clinical characteristics are non-specific, and the most common clinical manifestation is a rapidly enlarging (<6 months) neck mass, often combined with cervical lymph node enlargement or compression symptoms, such as dyspnea and dysphagia (2, 5), especially among patients with a background of HT (6). HT is a common autoimmune inflammatory disease of the thyroid gland and is widely recognized as a risk factor for PTL (22). Antonio et al. (6) reported that the prevalence of PTL combined with HT was 78.9%, and HT prevalence was significantly higher in patients with MALT lymphoma than in those with DLBCL. The ultrasound images of this patient indicated that HT could not be excluded and was speculated to be an important contributing factor to PTL.

Thyroid function in patients with thyroid MALT lymphoma varies, typically presenting as hypothyroidism, but it may also appear normal or hyperthyroid in the early stages. Changes in thyroid function may be closely related to HT and tumor infiltration. Thyroid MALT lymphoma exhibits a complex relationship with thyroid function. Hypothyroidism may lead to immunosuppression, increasing the risk of tumor development (23). Conversely, prolonged elevation of thyroid stimulating hormone levels may persistently stimulate thyroid cell proliferation, elevating the risk of malignant transformation (24). Thyroid function should be closely monitored and timely intervention should be implemented in the management of thyroid MALT lymphoma.

Ultrasonography is the primary imaging method for assessing the thyroid gland, playing a significant role in the diagnosis and differential diagnosis of thyroid-associated diseases. The ultrasonographic features of PTL include significantly hypoechoic lesions, linear or striped hyperechoic patterns, posterior echo enhancement, and slightly increased rich blood flow (25, 26). Additionally, some scholars have reported that the ultrasound manifestations of PTL vary by pathological types (27). MALT lymphoma typically presents as the diffuse type, while DLBCL tends to be more nodular. The diffuse type of MALT lymphoma in this case was consistent with the above conclusions. **Table 5** summarizes the imaging differences between MALT lymphoma and other primary thyroid malignancies.

**Table 5:** Differences of imaging characteristics between MALT lymphoma and other primary thyroid malignancies.

Item	Thyroid MALT lymphoma	Other primary thyroid malignancies
Ultrasound	Diffuse enlargement, homogeneous hypoechoic with unclear boundaries, rich blood flow	Nodular, heterogeneous hypoechoic with clear or unclear boundaries, often calcified, irregular blood flow
CT/MRI	Homogeneous low density/signal with unclear boundaries; mild homogeneous enhancement	Heterogeneous density/signal, heterogeneous enhancement, often calcified
[^18^F]FDG PET/CT	Homogeneous medium-high metabolism	No metabolism or low metabolism (DTC), heterogeneous hypermetabolism (ATC)
Lymph Node Metastasis	Rare	Common
Distant Metastasis	Rare	Relatively common (e.g., lung, bone, brain)

MALT, mucosa-associated lymphoid tissue; CT, computed tomography; MRI, magnetic resonance imaging; DTC, differentiated thyroid carcinoma; ATC, anaplastic thyroid carcinoma.

Ultrasound-guided CNB and fine needle aspiration (FNA) are widely used in the diagnosis of thyroid cancer; however, both methods make it relatively difficult to determine the pathologic subtype of PTL (28). The combination of immunochemical staining, flow cytometry, and polymerase chain reaction detection of immunoglobulin heavy chain gene rearrangement enhances the diagnostic value of CNB and FNA (29). In immunohistochemical staining, CD5, CD10, and CD23 are negative in MALT cases, whereas CD19, CD20, and CD45 are usually positive in DLBCL cases. Commonly used immunohistochemical markers are listed in **Table 6**. In this case, the patient underwent ultrasound-guided CNB combined with immunohistochemistry and gene rearrangement to accurately diagnose MALT lymphoma.

**Table 6:** Commonly used immunohistochemical markers.

Test	Type	Expected result
CD20	IHC	Positive
CD21	IHC	Positive
CD43	IHC	Positive/Negative
CD5	IHC	Negative
CD23	IHC	Negative
CD10	IHC	Negative
BCL2	IHC	Positive
BCL6	IHC	Positive
Cyclin D1 and SOX11	IHC	Negative
IgD	IHC	Negative
Congo Red	Histological staining	Positive/Negative

IHC, immunohistochemistry.

PTL typically shows hypermetabolic nodules or diffuse hypermetabolism in both lobes on [^18^F]FDG PET/CT (30, 31), with or without hypermetabolic lymph nodes. On CT, a diffuse mass is common. The long axis of the unilateral lesion is usually consistent with the long axis of the thyroid gland, and bilateral lesions usually grow along the contour of the gland. The internal density is often reduced, and cystic degeneration, necrosis, and calcification are rare (30). In this case, [^18^F]FDG PET/CT before treatment showed decreased thyroid density uniformity, expansive growth, and diffuse hypermetabolism, consistent with previous reports. However, the patient did not undergo PET/CT for post-treatment evaluation due to economic reasons. Our case demonstrates that in addition to primary thyroid cancer and chronic thyroiditis, PTL should also be considered when PET/CT imaging shows thyroid lesions with diffuse elevated [^18^F]FDG uptake (30, 32). Nakadate et al. reported that [^18^F]FDG PET/CT may be useful in distinguishing between PTL and chronic thyroiditis, as SUVmax values are significantly higher, and CT density is significantly lower in patients with PTL compared to those with chronic thyroiditis (30). This emphasizes that our case of primary thyroid MALT lymphoma displayed intense [^18^F]FDG accumulation on [^18^F]FDG PET/CT, whereas MALT lymphoma elsewhere in the body was typically only mildly or moderately metabolized (33–36). We believe this may be due to the partial overlap of the [^18^F]FDG uptake between MALT lymphoma and chronic thyroiditis. In addition, high [^18^F]FDG uptake implies that primary thyroid MALT lymphoma may have the potential to transform into aggressive lymphoma. [^18^F]FDG PET/CT imaging may aid in staging and evaluating the efficacy of thyroid lymphoma treatment.

**Figure 5:**
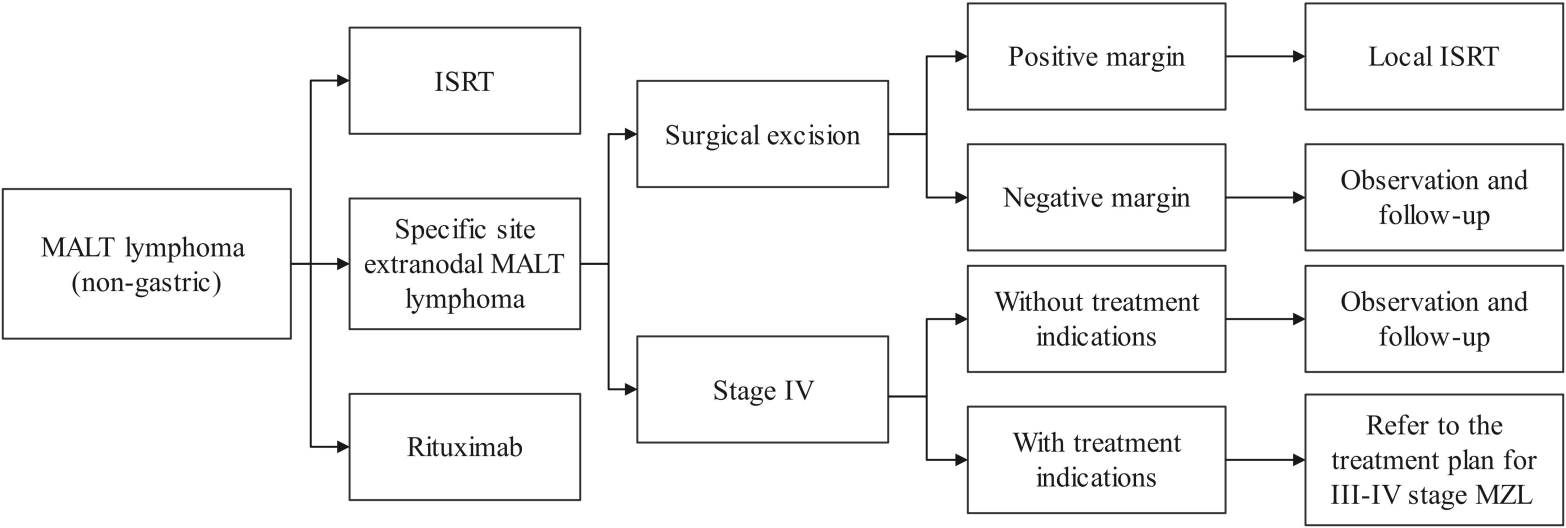
Treatment flowchart for extranodal MALT lymphoma (non-gastric). MALT, mucosa-associated lymphoid tissue; ISRT, involved site radiation therapy; MZL, marginal zone lymphoma.

At present, radiotherapy and chemotherapy are the main treatment strategies for PTL (4). Surgery is primarily used for biopsy or relief of compression symptoms. Blind surgical resection may result in permanent loss of thyroid function and increase the incidence of surgical complications. Rituximab is also a treatment option (37). The treatment flowchart for extranodal MALT lymphoma (non-gastric) is illustrated in **Figure 5**. In our case, the patient received R-CEOP chemotherapy, which demonstrated significant efficacy, and the patient achieved normal thyroid function. However, the side effects of chemotherapy, including myelosuppression, cardiotoxicity, and gastrointestinal reactions, limit its application in certain patients (38). Emerging alternatives such as targeted therapy and immunotherapy provide more options for patients with MALT lymphoma (39, 40). In the future, advancements in precision medicine, immunotherapy, and combination treatment strategies are expected to further enhance therapeutic outcomes and improve patients’ quality of life.

## Conclusion

In conclusion, it is important to consider the diagnosis of PTL in patients presenting with an enlarged neck mass and a history of HT, especially when accompanied by ‘B symptoms’ of lymphoma (fever, night sweats, weight loss, etc.). Ultrasonography and [^18^F]FDG PET/CT are helpful in the diagnosis and staging of PTL. Ultrasound-guided biopsy with pathological examination enables accurate subtype identification. Clinicians should avoid unnecessary thyroidectomy, which may lead to permanent thyroid function loss, and minimize the risk of surgical damage to adjacent organs and tissues, including the parathyroid gland. Future research should focus on optimizing patient management and treatment strategies to improve outcomes.

## Data Availability

The original contributions presented in the study are included in the article/supplementary material. Further inquiries can be directed to the corresponding author.
